# Retraction of: DeepCRISTL: deep transfer learning to predict CRISPR/Cas9 functional and endogenous on-target editing efficiency

**DOI:** 10.1093/bioinformatics/btad412

**Published:** 2023-07-10

Retraction of: DeepCRISTL: deep transfer learning to predict CRISPR/Cas9 functional and endogenous on-target editing efficiency, *Bioinformatics*, Volume 38, Issue Supplement_1, July 2022, Pages i161–i168, https://doi.org/10.1093/bioinformatics/btac218

Following article publication, the journal published a letter^1^ detailing flaws found in the evaluation methodology. The journal editors have investigated and are ultimately retracting the article as they feel readers can no longer rely upon the published findings.

^1^Letter to the editor: Testing on external independent datasets is necessary to corroborate machine learning model improvement, *Bioinformatics*, Volume 39, Issue 6, June 2023, btad327, https://doi.org/10.1093/bioinformatics/btad327

