# Correction to: “Retraction of: DeepCRISTL: deep transfer learning to predict CRISPR/Cas9 functional and endogenous on-target editing efficiency”

**DOI:** 10.1093/bioinformatics/btad562

**Published:** 2023-09-13

This is a correction to “Retraction of: DeepCRISTL: deep transfer learning to predict CRISPR/Cas9 functional and endogenous on-target editing efficiency”, *Bioinformatics*, Volume 39, Issue 7, July 2023, https://doi.org/10.1093/bioinformatics/btad412.

The retraction notice text has been updated, because we have subsequently discovered that the authors did not receive the journal’s communications to them asking them to address the flaws. This correction does not change the outcome or decision to retract.

